# Research trends and hotspots of post-stroke cognitive impairment: a bibliometric analysis

**DOI:** 10.3389/fphar.2023.1184830

**Published:** 2023-05-30

**Authors:** Xiansu Chi, Xueming Fan, Guojing Fu, Yue Liu, Yunling Zhang, Wei Shen

**Affiliations:** Xiyuan Hospital, China Academy of Chinese Medical Sciences, Beijing, China

**Keywords:** post-stroke cognitive impairment, hotspots, bibliometric, visualized analysis, literature review

## Abstract

**Background:** Post-stroke cognitive impairment (PSCI) is a major complication of stroke that affects more than one-third of stroke survivors, threatening their quality of life and increasing the risk of disability and death. Although various studies have described the etiology, epidemiology, and risk factors of PSCI, there are a limited number of comprehensive and accurate reports on research trends and hotspots in this field. Therefore, this review aimed to evaluate research trends, hotspots, and frontiers in PSCI using bibliometric analysis.

**Methods:** We screened the literature spanning 20 years in the Web of Science Core Collection: Science Citation Index Expanded (SCI-Expanded) database from 1 January 2003 to 31 December 2022. We included all eligible literature reports based on our comprehensive search strategy, inclusion criteria, and exclusion criteria. The analysis of annual publications, countries/regions, institutions, journals, co-cited references, and keywords was conducted using CiteSpace and VOSviewer, and the hotspots and major findings of PSCI were summarized.

**Results:** A total of 1,024 publications were included in this review. We found that the number of publications on PSCI increased annually. These publications were published in 75 countries or regions by over 400 institutions. Although Chinese institutions had the highest number of publications, their international influence was limited. The United States showed a strong influence in the field. The journal “Stroke” published the most publications (57) with a high impact factor and was considered the most co-cited journal. The most frequently cited references focused on the prevalence, incidence, neuropsychological assessment scales, criteria, and guidelines of PSCI. The strongest citation burst keywords for PSCI were “neurotrophic factor” and “synaptic plasticity”, which were regarded as research focuses and research hotspots, respectively.

**Conclusion:** This review provided a comprehensive summary of the literature of PSCI, identified the authoritative and frequently cited literature and journals, clarified the trends in PSCI research, and highlighted the hotspots in this field. Currently, studies on the mechanisms and treatment of PSCI are limited, and we hope that this review has effectively highlighted the research trajectory of PSCI and will lay the foundation for more innovative research in the future.

## 1 Introduction

According to the Global Burden of Disease Study 2019, stroke remains the second leading cause of death worldwide (Valery et al., 2021). The incidence of stroke is increasing, thus imposing a significant emotional and financial strain on families and resulting in a global economic burden of over $891 billion. In addition to focusing on reducing the incidence of stroke, it is also important to improve the prognosis after stroke (Owolabi et al., 2022). Cerebrovascular diseases are the second leading cause of acquired cognitive impairment. Vascular cognitive impairment (VCI) refers to the clinical syndrome of impairment in at least one area of cognition caused by cerebrovascular diseases and their risk factors (O'Brien et al., 2003; Chinese Society of Neurology, 2011; Gorelick et al., 2011; Skrobot et al., 2018). Post-stroke cognitive impairment (PSCI), which constitutes the main part of VCI, has only received increased attention in recent years (Pantoni and Salvadori, 2021).

PSCI is a major complication of stroke that affects more than one-third of stroke survivors (Mijajlović et al., 2017) and manifests as cognitive impairment within 3–6 months after the onset of stroke. Depending on the degree of cognitive impairment, PSCI can be divided into post-stroke cognitive impairment no dementia and post-stroke dementia (Quinn et al., 2021a; Rost et al., 2022). PSCI highlights the causal relationship between stroke events and cognitive impairment, indicating that stroke events directly lead to cognitive impairment. A previous study found that post-stroke dementia occurs in approximately 10% of stroke survivors after the first stroke and 30% after the recurrent stroke (Pendlebury and Rothwell, 2009). PSCI is characterized by persistent impairment in one or more core cognitive domains, such as attention, memory, executive function, language, and visuospatial ability (Rost et al., 2022), and significantly increases the risk of death, disability, and depression within 5 years of stroke onset (Obaid et al., 2020).

Since VCI is a general term, it lacks specificity in mechanism, prognosis, and treatment (van der Flier et al., 2018; Biesbroek and Biessels, 2023). As a subtype of VCI, PSCI has a characteristic feature of relatively fixed brain imaging results, and a clear causal relationship between stroke and cognitive function. Therefore, if we narrow the scope of research and dig into the research trends and hotspots of PSCI clearly, the results will not be interfered by other subtypes and can be very valuable. As a unique subgroup of VCI, PSCI highlights the event of stroke as a trigger point and important tip points that can attract sufficient attention from patients, families, and doctors. Compared with other subtypes of VCI, PSCI can be diagnosed in a timely manner, and can get early intervention and treatment, which provide more benefits and value to the society. Therefore, more research on PSCI is necessary and valuable. Over the past 20 years, PSCI has become an interesting research direction, with several studies published annually on its etiology, epidemiology, and risk factors. However, despite this exponential growth, effective and targeted strategies available to manage PSCI remain limited in the clinic. Therefore, it is crucial to include the comprehensive and accurate PSCI literature, analyze current research trends, and identify hotspots to guide future research on PSCI.

Bibliometric analysis uses quantitative methods to describe, evaluate, and monitor published research, providing insights into the structure, social networks, and thematic interests of the field through citation and keyword analysis (Zupic and Čater, 2014).

In this review, we aim to conduct a comprehensive screening of publications on PSCI and use bibliometric analysis to reveal the evolutionary trends and major findings in the field. This review provides a deeper understanding of PSCI and summarizes the key developments, research frontiers, and hotspots in PSCI research over the past 20 years.

## 2 Materials and methods

### 2.1 Search strategy

The term VCI was introduced in the early 21st century (van der Flier et al., 2018), and the nomenclature for PSCI has recently been standardized. However, since various nomenclatures are used for PSCI, we took full consideration of the possible alternative descriptions of PSCI when summarizing our search strategies. We expanded the scope of the search to ensure that the literature with different descriptions of PSCI could be retrieved. The specific search strategies are recorded in Supplementary Materials S1. The studies were retrieved from the SCI-expanded database of the Web of Science Core Collection. Given the tremendous development in the PSCI field, we chose a 20-year period, from 1 January 2003 to 31 December 2022 for our analysis. Publications were limited to articles and reviews.

### 2.2 Inclusion criteria

Publications that included patients or animals with a confirmed diagnosis of PSCI or publications, in which patients or animals had a basic diagnosis of stroke and showed cognitive impairment, were both included. Additionally, the research content should focus on epidemiology (including demographic factors and risk factors), mechanisms, pathophysiology, diagnosis, screening, prevention, management, or treatment of PSCI.

### 2.3 Exclusion criteria

Completely irrelevant literature studies and those that focused on PSCI relevant disease but the research content only focused on the epidemiology (demographic factors and risk factors), mechanisms, diagnosis, pathophysiology, screening, prevention, management, or treatment of other relevant diseases but not PSCI were excluded.

**FIGURE 1:**
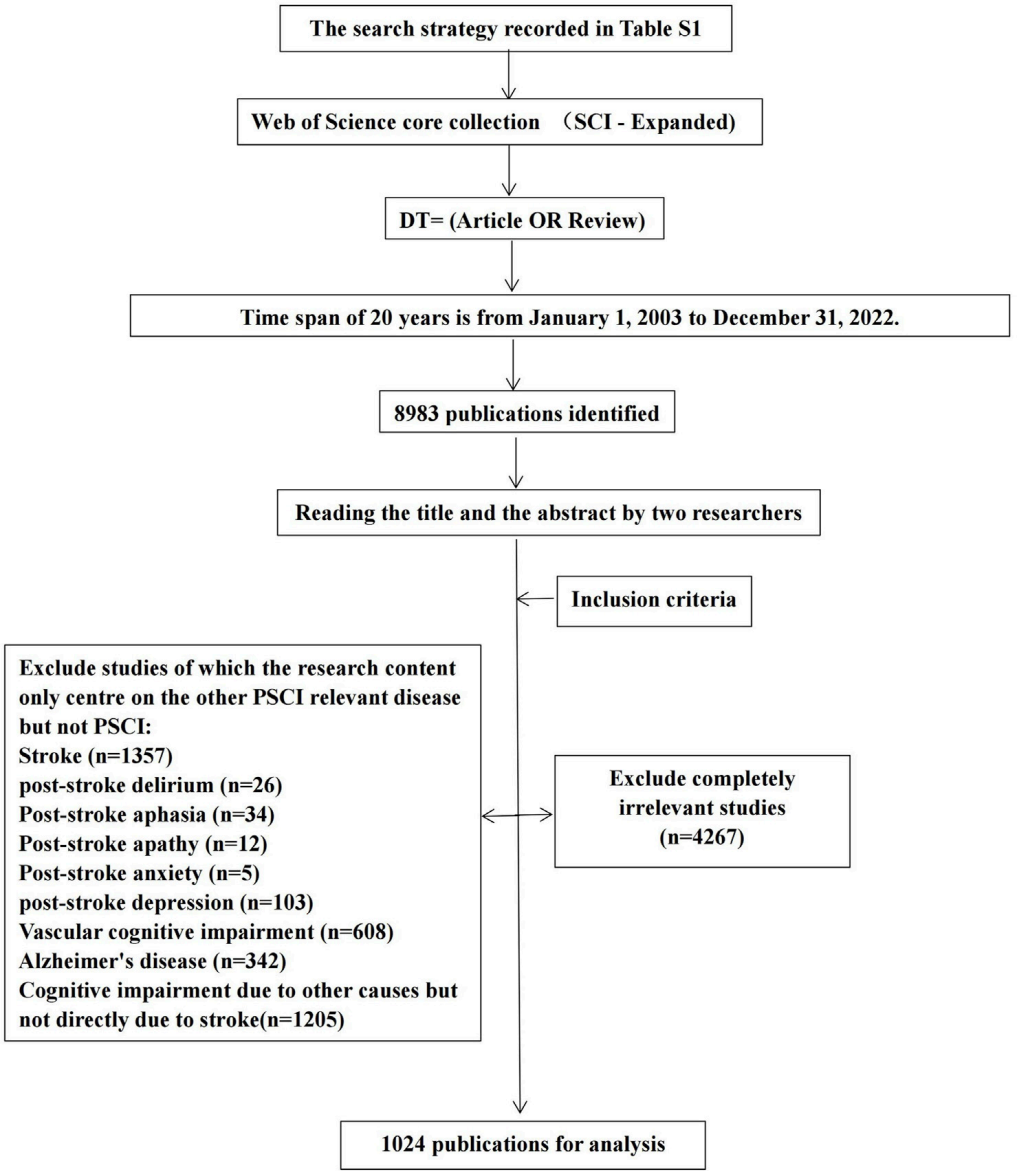
Flow chart of literature selection.

All publications were independently screened by two researchers, who specialized in encephalopathy, based on the inclusion and exclusion criteria by reading the titles and abstracts. Two researchers jointly confirmed the results, and the third researcher resolved any disagreements. Figure 1 depicts the screening process for publications, and the reasons for exclusion are recorded in Supplementary Material S2.

### 2.4 Analytical tool

The full record and cited references of the publications were downloaded, and visual analysis was performed using CiteSpace (version 6.1. R6; Drexel University, PA, United States) and VOSviewer (version 1.6.17; Leiden University, Netherlands) software applications. ArcMAP (version 10.8; Esri, CA, United States) was used to geovisualize the number of publications.

## 3 Results

### 3.1 Publication output

**FIGURE 2:**
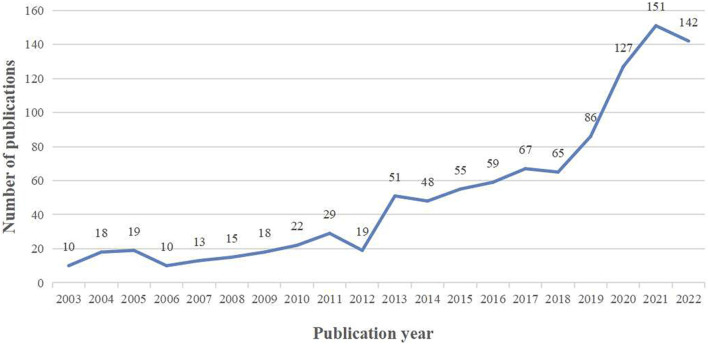
Annual output of post-stroke cognitive impairment (PSCI) over the past 20 years.

The output of research related to PSCI from 2003 to 2022 showed an overall year-on-year increase (Figure 2). A total of 1,024 publications met our inclusion criteria, consisting of 905 (88.37%) articles and 119 (11.63%) reviews. The PSCI literature was broadly divided into two phases that reflected the development trends in this field. In the first phase, from 2003 to 2012, the annual number of studies was roughly constant, ranging from 10 to 29 articles per year, with 173 publications in total. In the second phase, from 2013 to 2022, the annual number of literature reports grew rapidly, with a total of 851 publications. In particular, a large number of studies were published from 2020 to 2022, highlighting the increasing attention from researchers in recent years.

### 3.2 Country or region analysis

**FIGURE 3:**
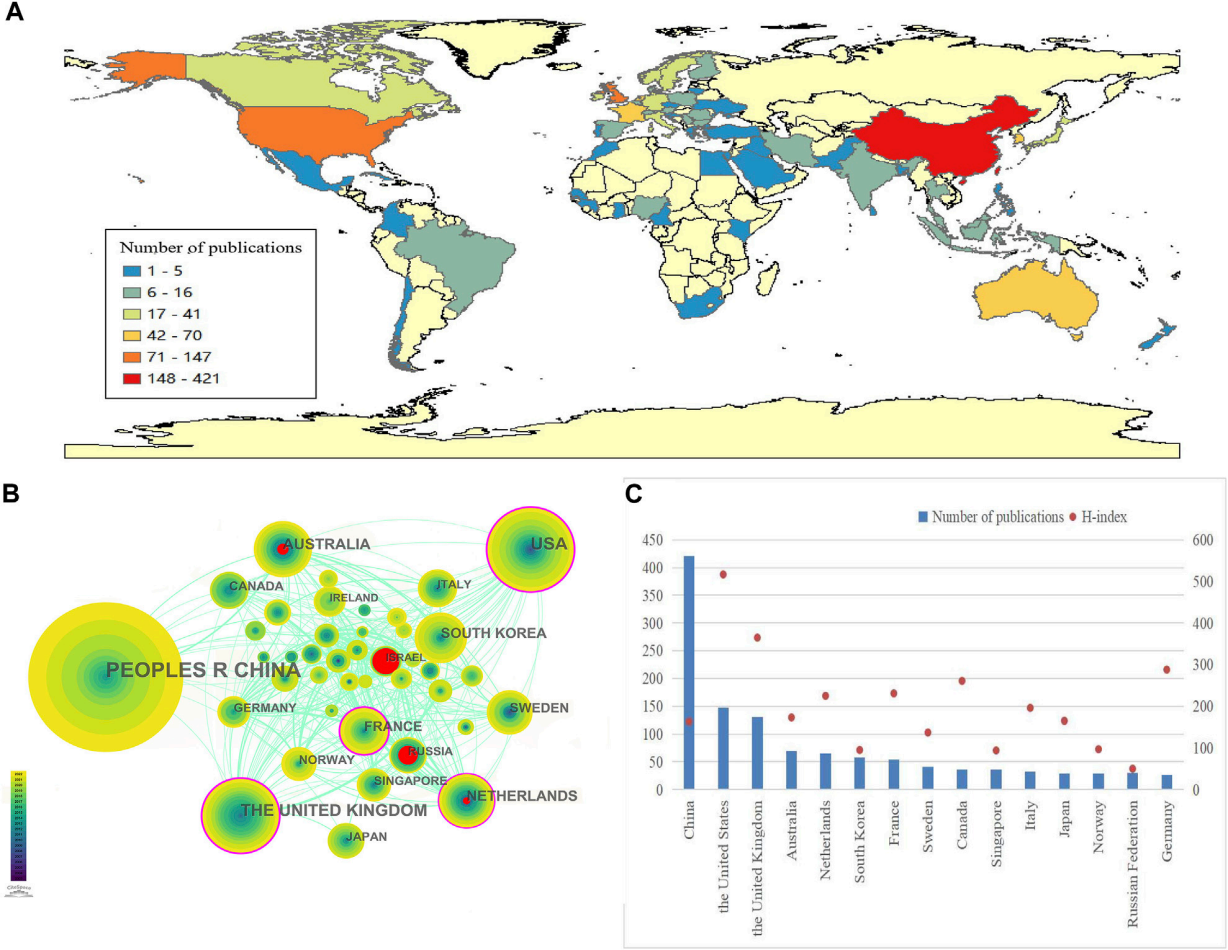
Geographical distribution and network of countries/regions in post-stroke cognitive impairment: **(A)** Geographical heatmap of publications; **(B)** co-occurrence network of countries/regions; and **(C)** number of PSCI publications in the top 15 countries or regions and their H-index.

PSCI-related research was conducted in 75 countries or regions from 2003 to 2022. The breakdown of publications by country is shown in Figure 3A. In Asia, China ranked first with 421 publications, which accounted for more than one-third of the total number of publications, making it the core research country in this field. The United States (U.S.) in North America and the United Kingdom (U.K.) in Europe followed with 147 and 131 publications, respectively. Twenty-three other countries (30.7% of the total) published more than 10 publications. Overall, Asia published the greatest number of PSCI-related studies (665), followed by Europe (542) and North America (185). To some extent, the number of publications in different states reflected the attention and concern of researchers regarding PSCI in those regions.

The country or region collaboration network was constructed using CiteSpace (Figure 3B). Each node represented a country. Nodes with purple rings represented high centrality (centrality ≥0.1), indicating that they were considered to be of high importance and influence (Synnestvedt et al., 2005). Countries such as the U.S., the U.K., the Netherlands, and France showed higher centrality in this field. Nodes with red annual rings indicated an increase or rapid continual increase in the annual frequency of citations. Israel, Russia, Australia, and the Netherlands showed the strongest citation bursts within a certain period, indicating that these countries have a high scholarly activity and their research studies have been widely followed by scholars.

The H-index is an indicator of the scientific impact of a scholar or country, reflecting that a scholar/country has published H publications and that each publication has been cited at least H times by other publications (Xia et al., 2021). By analyzing the H-index in the field of cognitive neuroscience, we found that that the U.S. (517) had the highest score and the overall scientific research impact, followed by the U.K. (365), Germany (288), and Canada (261). Despite China ranking first in the number of publications, its H-index was 163, indicating a limited academic influence and a need for ongoing efforts to improve the research quality. The top 15 countries or regions in the number of PSCI publications and their corresponding H-index values are displayed in Figure 3C.

### 3.3 Institutional analysis

**FIGURE 4:**
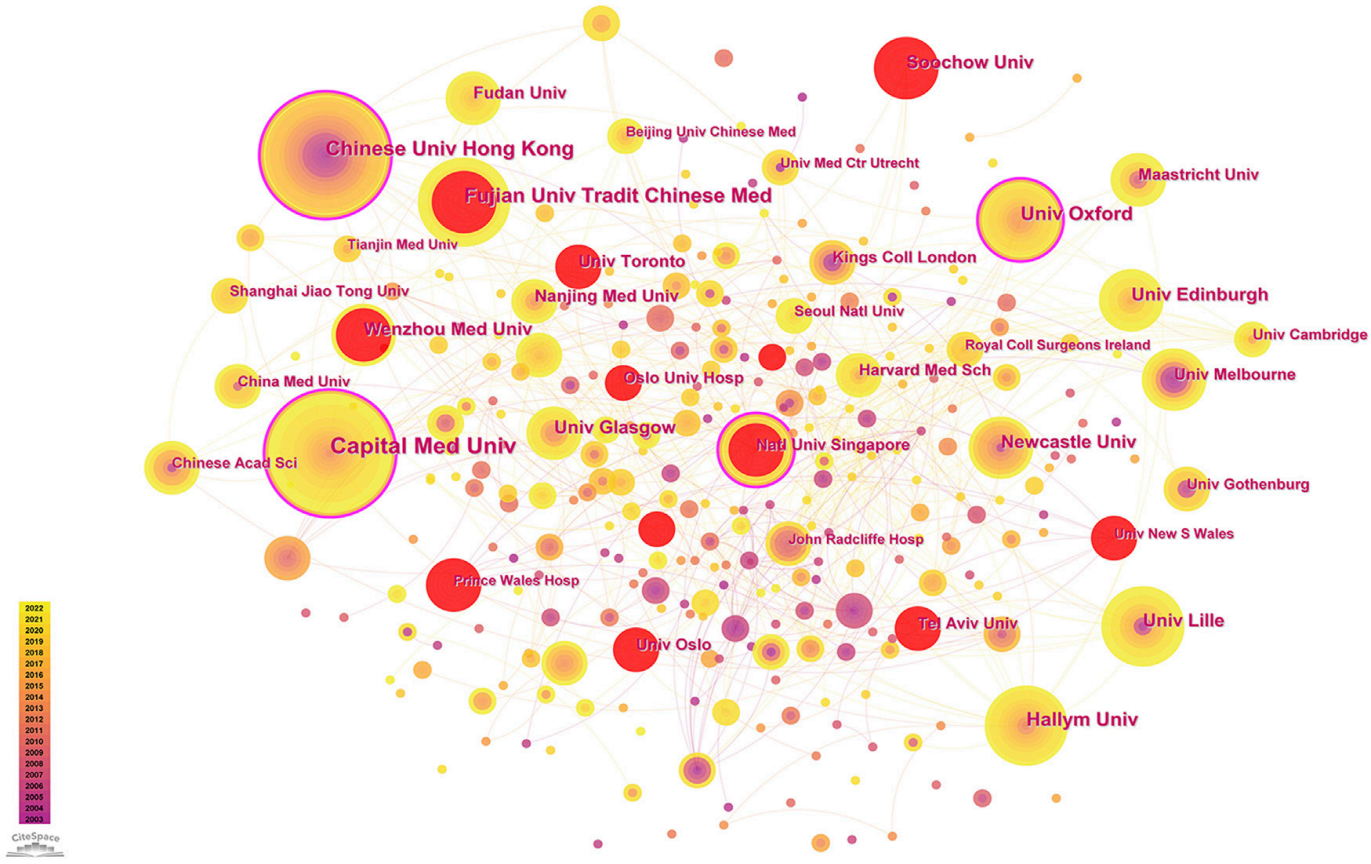
Network of institutions engaged in PSCI.

Between 2003 and 2022, more than 400 institutions conducted research on PSCI. An institutional collaboration network was established using CiteSpace (Figure 4). Among these, the Capital Medical University (39 publications), the Chinese University of Hong Kong (26 publications), and the Fujian University of Traditional Chinese Medicine (26 publications) in China were the top three institutions in terms of the number of published studies with high centrality, indicating their extensive interest and strong influence in this field. Other institutions that published more than 20 PSCI-related literature studies include Oxford University (25 publications) in the U.K., Hallym University (22 publications) in Korea, Tel Aviv University (22 publications) in Israel, the University of Glasgow (22 publications) in the U.K., and the National University of Singapore (21 publications) in Singapore. Furthermore, the Fujian University of Traditional Chinese Medicine in China (26 publications), Tel Aviv University in Israel (22 publications), Wenzhou Medical University in China (20 publications), the University of Toronto in Canada (20 publications), and Soochow University in China (19 publications) showed rapid growth over time and potential breakthroughs within a certain period.

### 3.4 Journal analysis

**FIGURE 5:**
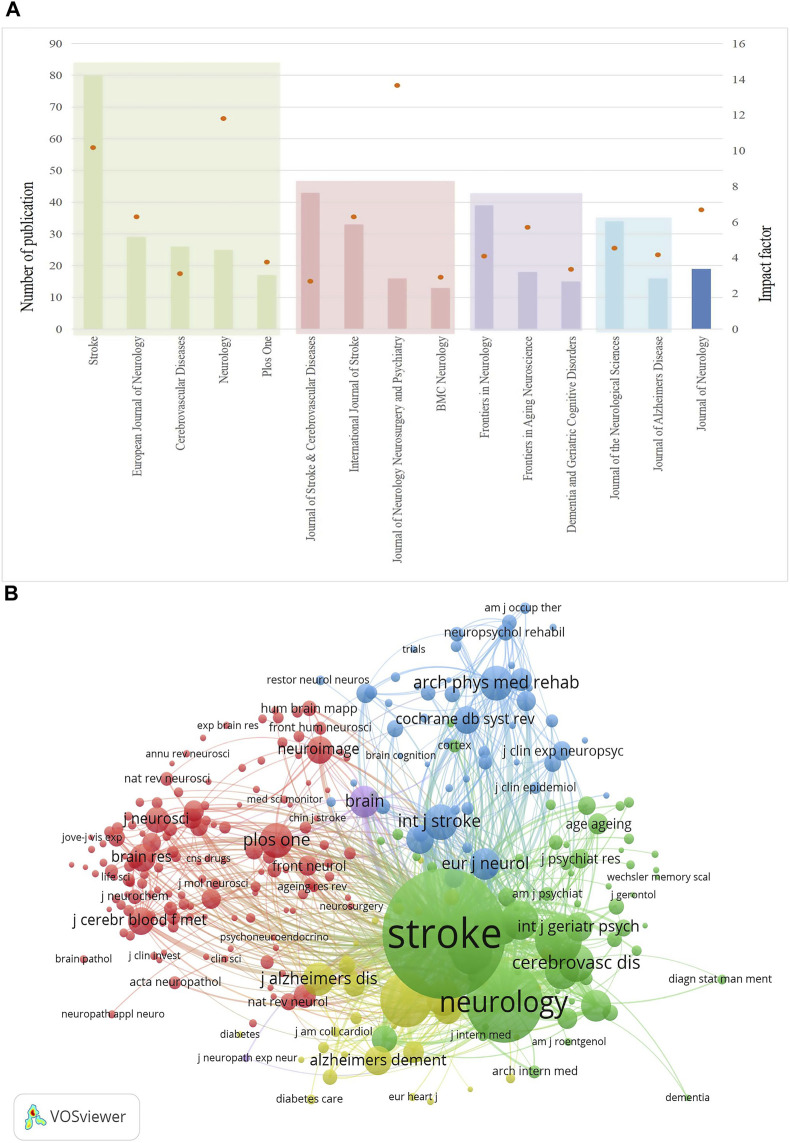
Journals that published PSCI research. **(A)** Top 15 journals published PSCI research publications and their impact factor. **(B)** Network of co-cited journals.

Between 2003 and 2022, PSCI research was published in 355 academic journals. The top 15 journals with the highest outputs and their respective impact factors are shown in Figure 5A and presented in Table 1. Among them, *Stroke* ranked first with 57 publications (5.62%), followed by the *Journal of Stroke & Cerebrovascular Diseases* with 43 publications (4.23%) and *Frontiers in Neurology* with 38 publications (3.46%). After analyzing the top 15 academic journals, we found that five were from the U.S., four from the U.K., three from Switzerland, two from the Netherlands, and one from Germany. The following three journals, with the impact factor (IF) greater than 10, were deemed high-impact journals in this field: *Journal of Neurology, Neurosurgery, and Psychiatry* (IF = 13.654), *Neurology* (IF = 11.8), and *Stroke* (IF = 10.17). They are widely recognized for their high influence in PSCI research.

**TABLE 1:** Top 15 journals in the post-stroke cognitive impairment (PSCI) field.

Rank	Journal	Publication (%)	Country/region	Impact factor (2021)	Quartile
1	Stroke	57	The United States	10.17	Q1
2	Journal of Stroke and Cerebrovascular Diseases	43	The United Kingdom	2.677	Q4
3	Frontiers in Neurology	38	Switzerland	4.086	Q3
4	Journal of the Neurological Sciences	22	The Netherlands	4.533	Q3
5	Neurology	19	The United States	11.8	Q1
6	Frontiers in Aging Neuroscience	18	Switzerland	5.702	Q2
7	PLOS ONE	17	The United States	3.752	Q3
8	International Journal of Stroke	15	The United Kingdom	6.948	Q2
9	Dementia and Geriatric Cognitive Disorders	15	Switzerland	3.346	Q4
10	Journal of Alzheimer’s Disease	15	The Netherlands	4.160	Q3
11	Cerebrovascular Diseases	14	The United States	3.104	Q3
12	Journal of Neurology, Neurosurgery, and Psychiatry	14	The United Kingdom	13.654	Q1
13	Journal of Neurology	14	Germany	6.682	Q2
14	European Journal of Neurology	13	The United States	6.288	Q3
15	BMC Neurology	13	The United Kingdom	2.903	Q4

A co-citation can reveal influential journals in a particular field by measuring the frequency with which two journals are cited together in the same publication (Zhang et al., 2022). In this review, journal co-citations were analyzed using VOSviewer, and the results showed that *Stroke* (4,763)*, Neurology* (2,040)*, Lancet Neurology* (1,043)*,* and *Journal of Neurology, Neurosurgery, and Psychiatry* (855) were the top four co-cited journals (Figure 5B), indicating their superior scholarly performance in the field of PSCI.

**FIGURE 6:**
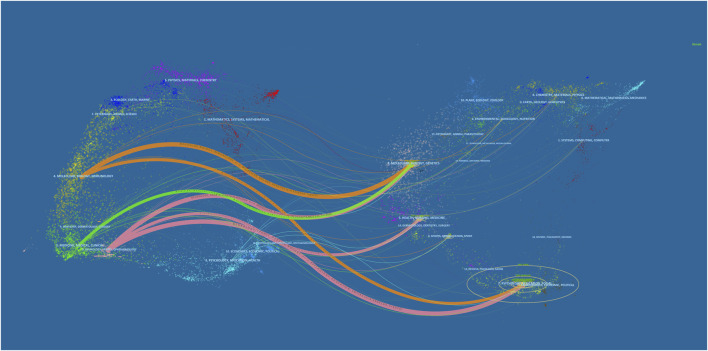
Dual-map overlay of journals related to PSCI research.

The dual-map overlay atlas is a tool that visually links citing journals on the left with cited journals on the right to specifically reveal the citation relationship and subject distribution of the literature (Zhang et al., 2022). The disciplines are distinguished by colored lines in Figure 6. The citing journals belong mainly to molecular biology, immunology, medicine, medical, clinical, neurology, sports, and ophthalmology journals. However, the cited references are published in molecular biology, genetics and psychology, education, social molecular, , immunology, health, nursing, medicine, psychology, and social journals. Overall, PSCI was found to be closely connected to basic scientific, clinical, and social subjects. In the future, it is recommended to further strengthen the connection of multi-subjects.

### 3.5 Co-cited references and references with citation bursts

The top 10 co-cited references are shown in Table 2. Among them, each reference was co-cited at least 84 times. Five references were co-cited more than 100 times. After analyzing these five highly co-cited references, we found that these publications primarily focused on the prevalence, incidence, neuropsychological assessment scales, criteria, and guidelines for PSCI.

**TABLE 2:** Top 10 co-cited references in the post-stroke cognitive impairment field.

Rank	Reference	Co-citation	Year
1	Prevalence, incidence, and factors associated with pre-stroke and post-stroke dementia: a systematic review and meta-analysis	258	2009
2	The Montreal Cognitive Assessment (MoCA): a brief screening tool for mild cognitive impairment	194	2005
3	“Mini-mental state”: a practical method for grading the cognitive state of patients for the clinician	178	1975
4	National Institute of Neurological Disorders and Stroke-Canadian Stroke Network vascular cognitive impairment harmonization standards	132	2006
5	Vascular contributions to cognitive impairment and dementia: a statement for healthcare professionals from the American Heart association/American Stroke association	107	2011
6	Post-stroke dementia	97	2005
7	Post-stroke cognitive impairment: epidemiology, mechanisms, and management	89	2014
8	Cognitive impairment after stroke: frequency, patterns, and relationship to functional abilities	89	1994
9	Post-stroke dementia: a comprehensive review	88	2017
10	Classification of subtype of acute ischemic stroke. Definitions for use in a multicenter clinical trial. TOAST. Trial of Org 10,172 in Acute Stroke Treatment	84	1993

In addition to having the highest number of the most citations, the literature *Prevalence, incidence, and factors associated with pre-stroke and post-stroke dementia: a systematic review and meta-analysis* had the highest centrality (centrality = 0.33), indicating its significant contribution to this field.

**FIGURE 7:**
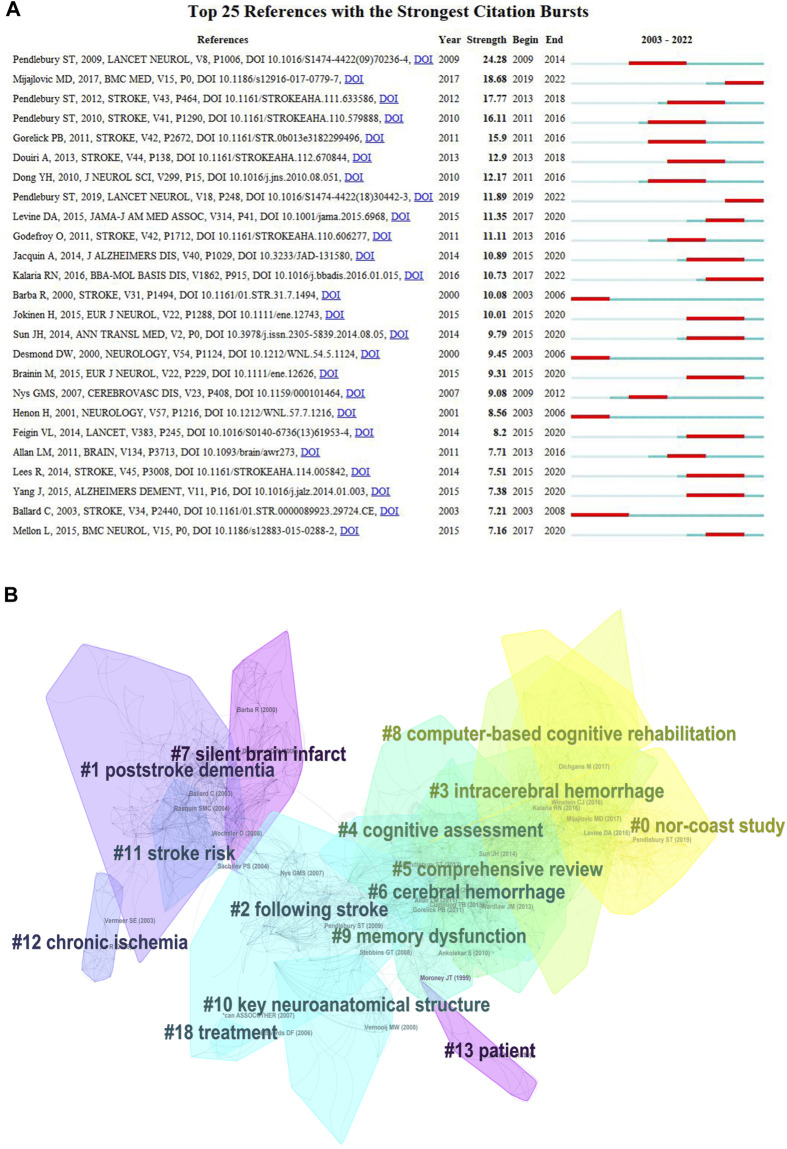
Top 25 reference with the strongest citation bursts and cluster diagrams of references in PSCI research. **(A)** Top 25 references with the strongest citation bursts. **(B)** Cluster results of the references in PSCI research.

Burst detection can be utilized to identify references that have received collective attention during a specific period. The top 25 references with the strongest citation bursts are shown in Figure 7A. Upon analyzing these references, we found that studies authored by Pendlebury were widely cited from 2009 to 2022, covering topics such as the incidence and prevalence of post-stroke dementia, as well as the underestimation of cognitive assessment scales.

Using CiteSpace, the co-cited references were clustered and found to be mainly related to the Norwegian Cognitive Impairment After Stroke (Nor-COAST) study, post-stroke dementia, stroke, intracerebral hemorrhage, cognitive assessment, comprehensive reviews, cerebral hemorrhage, silent brain infarction, computer-based cognitive rehabilitation, memory dysfunction, key neuroanatomical structures, stroke risk, chronic ischemia, and patients (Figure 7B).

### 3.6 Keyword analysis

**FIGURE 8:**
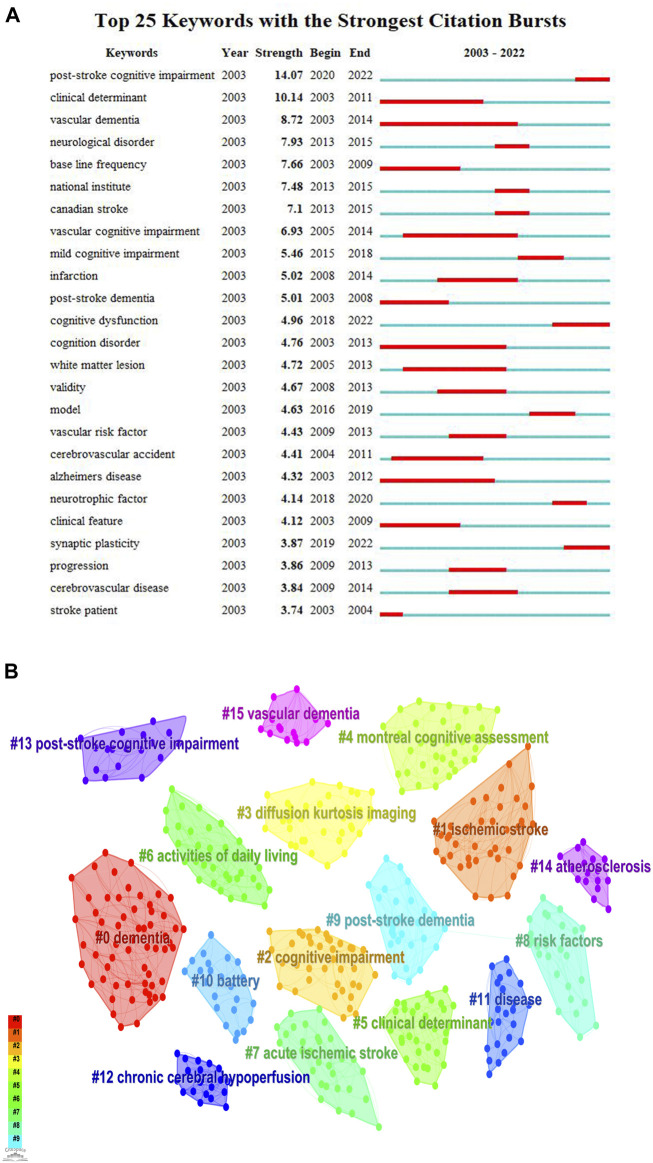
Top 25 keywords with the strongest citation bursts and cluster diagrams of keywords in PSCI research. **(A)** Top 25 keywords with the strongest citation bursts. **(B)** Cluster results of the keywords in PSCI research.

Burst keywords indicate the widespread occurrence of keywords at certain stages and are sorted according to their burst intensity and the year the burst begins and ends. The top 25 keywords with the strongest citation burst are shown in Figure 8A. The most frequently mentioned keywords include stroke, vascular dementia (VaD), vascular cognitive impairment, Alzheimer’s disease (AD), PSCI, post-stroke dementia, and cognitive disorders. These diseases are closely linked to PSCI and necessitate careful differential diagnosis. Apart from these clinically relevant diseases, keywords with the longest burst duration include clinical determinant, white matter lesion, validity, model, neurotrophic factor, clinical feature, and synaptic plasticity. The keywords “neurotrophic factor” and “synaptic plasticity” showed the strongest citation bursts between 2017 and 2022 and can be regarded as the indicators of researcher attention or emerging research hotspots. As shown in Figure 8B, we clustered the keywords into 16 categories and analyzed them to better comprehend the PSCI research trajectories in the literature. Excluding related diseases, the categories encompass diffusion kurtosis imaging, the Montreal Cognitive Assessment (MoCA), clinical determinants, activities of daily living, risk factors, and battery. Diffusion kurtosis imaging scans are assessment tools used to detect microstructural alterations in the white matter (He et al., 2022), and it is reported that neuroanatomical lesions caused by stroke in key regions, such as the hippocampus and white matter lesions, may be one of the mechanisms responsible for the development of PSCI (Sun et al., 2014). The risk factors and common neuropsychological assessment scales for PSCI need to be further summarized and investigated.

## 4 Discussion

### 4.1 General information

In terms of the annual publication output, the trend in the number of publications from 2003 to 2022 has undergone two phases of growth. From 2003 to 2012, the number of publications grew slowly in a flat situation and lacked breakthroughs. During this phase, PSCI was named differently and was not sufficiently considered. From 2013 to 2022, the annual number of studies grew rapidly. During this time, the statement “post-stroke dementia is an indispensable part of stroke care” was made on World Stroke Day in 2015; and the concept of “cognitive function reestablishment should be integrated with stroke intervention strategies” was established at the International Stroke Conference in February 2016. These major events reflect that researchers have paid more attention to PSCI. In particular, there was a rapid increase in the number of publications from 2020 to 2022, which may be related to two publications: the PSCI joint guidelines published by the European Stroke Organization and European Academy of Neurology (Quinn et al., 2021b), and the Chinese Expert Consensus 2021 on PSCI (Wang et al., 2021). These publications provide evidence-based recommendations for clinicians to better prevent, diagnose, and treat PSCI, and highlight the areas where robust evidence is still lacking, thereby pointing out the way for subsequent research.

During the early stages of stroke recovery, rehabilitation of limb motor function typically receives significant research attention. However, as the treatment process progresses, cognitive impairment plays a vital role in the overall patient recovery progress (Zhang et al., 2020). Therefore, it is essential to increase attention to PSCI.

With regards to the national and institutional cooperation networks involved in the research on PSCI, it is worth noting that while this topic has been the focus of more than 400 institutions and 75 countries/regions, including China and the U.S., the majority of nations are yet to produce publications related to this field. This phenomenon may be attributed to two primary reasons. On one hand, some developing countries continue to place their research emphasis on reducing the mortality rates of stroke, which is regarded as the second leading cause of death (Valery et al., 2021). On the other hand, the Netherlands and France, which have high centrality in contacts with other countries, and Australia, which had the strongest citation burst over a certain period, have reported that over 45% of stroke survivors suffer from PSCI. This higher incidence may account for the increased concern about PSCI in these countries.

As for institutional cooperation, Chinese universities have conducted more research on PSCI. However, their international influence still requires improvement. The large number of publications in China with low quality may be attributed to several reasons. First, while China has published a significant number of articles, there are fewer original articles and more review articles, making it difficult to achieve innovative discoveries. Second, there are issues with the science and technology evaluation system, where the number of published articles and IF are used as the standard for career promotion and performance consideration, leading to a focus on quantity over quality. This, in turn, leads to research that is carried out purely for career promotion purposes and lacks meaningful outcomes. Third, there is a lack of regulatory policies for article publication in China, leading to publications that have not undergone strict checks and may contain several errors. Finally, a large number of imitations and instances of plagiarism may also lead to a significant number of articles with low quality. Therefore, it is recommended that policies should be developed at the national level to encourage innovative research and prevent the overvaluation of SCI. In addition, career promotion and evaluation should prioritize the quality and innovation of articles over their quantity. At the individual level, strict supervision should be conducted before publishing an article, and a down-to-earth attitude should be adopted with no plagiarism or imitations. In addition, coordination and cooperation among institutions, especially between institutions in different countries, remain insufficient. Currently, the collaboration between countries indicates a positive relationship. For example, the joint guidelines on PSCI published by the European Stroke Organization and the European Academy of Neurology (Quinn et al., 2021b) have facilitated intercollaboration between countries and institutions to some extent and improved the evidence for the management of PSCI.

As a high IF journal, *Stroke* has published the highest number of PSCI publications and is considered the top co-cited journals. Research studies focused on PSCI in this journal are therefore regarded as more authoritative, highly instructive, and well recognized in the field. The prevalence, incidence, neuropsychological assessment scales, criteria, and guidelines for PSCI are regarded as the core topic of these most co-cited references. Several studies have been conducted on PSCI in Norwegian patients, collectively known as the Nor-COAST study, which has provided abundant clinical data on PSCI.

### 4.2 Hotspots and major findings

In this review, we have aimed to provide a comprehensive summary of the hotspots and major findings in the field of PSCI, including the diseases related to PSCI, risk factors, common neuropsychological assessment scales, and animal models. Our goal in conducting this review was to assist researchers in avoiding outdated and repetitive research and to optimize the use of project finding by incorporating innovative research that aligns with the highlighted research hotspots. We are hopeful that this review will serve as a valuable resource for researchers seeking to advance the field of PSCI and improve the quality of research in this area.

#### 4.2.1 PSCI and related disease

AD is a neurodegenerative disease that causes severe atrophy of the cerebral cortex, leading to the narrowing of the gyrus and widening of the sulcus (Martin, 2010). The pathogenesis of AD is believed to be related to cholinergic, *β*-amyloid, tau, and oxidative stress hypotheses. AD is characterized by the deposition of insoluble *β*-amyloid peptides and the formation of neurofibrillary tangles composed of abnormal tau protein (Bloom, 2014; Fakhoury, 2018). VCI is the second leading cause of cognitive impairment (Hort et al., 2019). It is characterized by an impairment in at least one cognitive domain resulting from subclinical vascular brain injury or clinical stroke caused by cerebrovascular dysfunction, and it ranges from mild cognitive impairment to dementia, including varying degrees of cognitive deficit due to mixed pathologies (Gorelick et al., 2011; Skrobot et al., 2018). Research has found that approximately one-third of patients with VaD may have coincidental AD pathology (Gorelick and Nyenhuis, 2013) and that amyloid plaques and neurofibrillary tangles are more likely to cause AD if accompanied by vascular risk (Rabin et al., 2018). Moreover, plasma osteopontin, brain angiotensin type 2 receptors, and 20-hydroxyeicosatetraenoic acid have been confirmed as the common potential targets and biomarkers of AD and VCI (Ahmed and Ishrat, 2020; Chai et al., 2021; Gonzalez-Fernandez et al., 2021).

PSCI, a type of VCI, is a major complication of stroke that mediates a range of syndromes, from mild cognitive impairment to dementia, with emphasis on stroke events triggering cognitive dysfunction (Zhang and Bi, 2020). Studies have revealed a significant overlap in the neuropsychiatric features of AD, VCI, and PSCI (Kim et al., 2022).

Currently, no effective drug therapies for PSCI or VCI have been approved by the Food and Drug Administration (FDA) (Shim, 2014; Kim et al., 2020). However, despite our limited understanding of the mechanism of PSCI, due to the overlapping neuropathological mechanisms, drugs approved by the FDA for AD, such as donepezil, cholinesterase inhibitors, and non-competitive N-methyl-D aspartic acid receptor antagonists, are effectively administered to treat PSCI and VCI (Wang et al., 2021). Therefore, extensive research is needed to explore the pathogenesis and therapeutic targets of PSCI and to develop therapies specific to PSCI.

#### 4.2.2 Risk factors of PSCI

PSCI is thought to be associated with various factors, including demographic, stroke-related, and vascular risk factors (Huang et al., 2022). Older age has been linked to a higher risk of PSCI, VCI, and AD (Rodríguez García and Rodríguez García, 2015). Individuals with a lower education level have a 3.03-fold higher likelihood of developing PSCI than those with a higher level of education. It is also hypothesized that individuals with higher education have greater cognitive reserve, which may delay the onset of cognitive impairment through compensatory mechanisms (Duda et al., 2014). The higher prevalence of PSCI in females may be attributed to differences in stroke susceptibility and average life expectancy between sexes, with females being more prone to cardiac embolic stroke and males more likely to have a lacunar stroke (Nys et al., 2007). Additionally, a study showed that all the three countries with the highest number of PSCI publications had a high prevalence of PSCI. In Shanghai, China, the prevalence of PSCI was 53.1% at 6–12 months after stroke; in South Texas, U.S., it was 27.6% in men and 35.6% in women at 3 months after stroke; and in Edinburgh, U.K., it was 44% at 3 years after stroke (Huang et al., 2022). We speculate that the high incidence of PSCI in these countries has motivated researchers to devote more attention to this condition. Furthermore, pre-stroke cognitive impairment or dementia could increase the risk of mortality and cognitive impairment (Hommel et al., 2015), and thus, pre-stroke cognitive impairment, possibly co-existing with a neurodegenerative pathology, may contribute to the development of PSCI (Liu et al., 2015).

Various stroke-related factors, such as previous stroke history and the location of stroke lesions, have been found to impact the risk of PSCI. Lesions in different strategic brain regions, including the basal ganglia, thalamus, splenium of the corpus callosum, posterior internal capsule, and cingulate cortex, have been linked to PSCI. Particularly, infarcts in the left frontotemporal lobe, right parietal lobe, and left thalamus have been strongly linked to PSCI development (Weaver et al., 2021). Medial temporal lobe atrophy and hippocampal atrophy are considered as key biomarkers of AD and are also associated with VaD and post-stroke dementia. The mechanism of atrophy involves chronic cerebral hypoperfusion that alters the synaptic connections between neurons (Kalaria and Ihara, 2017). White matter changes have been found to be significantly correlated with the medial temporal lobe atrophy (Akinyemi et al., 2015), and changes in the frontal and parieto-occipital regions are associated with the hippocampal atrophy (de Leeuw et al., 2004). White matter hyperplasia is often observed in patients with cognitive impairment. Magnetic resonance imaging is used to measure this, and diffusion kurtosis imaging can also be used as an assessment tool to detect microstructural alterations in the white matter of patients with PSCI (He et al., 2022). This may explain why diffusion kurtosis imaging is often included as the main keyword category.

The risk factors for vascular injury include atrial fibrillation, carotid artery disease, chronic kidney disease, diabetes, hypertension, alcohol consumption, and smoking. Atrial fibrillation, in particular, has been closely linked with cardiogenic stroke (Sachdev et al., 2006; Jung et al., 2010), which can exacerbate the infarct size, leading to PSCI (Chander et al., 2017). In addition, the progressive development of carotid atherosclerotic calcification and increased stiffness in the carotid arteries can cause decreased cerebral blood flow and breakdown of neurovascular coupling, ultimately accelerating cognitive dysfunction (Jefferson et al., 2018). Patients with chronic kidney diseases are prone to have memory and visuospatial impairment after stroke, whereas those with type 2 diabetes mellitus experience the main impairment in memory, executive function, and attention 2 years after the stroke onset (Ben Assayag et al., 2017). Moreover, patients with both type 2 diabetes mellitus and chronic kidney diseases have a four-fold increased risk of cognitive dysfunction. Smoking is recognized as a risk factor for cognitive deficit and cerebrovascular events (Virdis et al., 2010). Alcohol consumption has also been associated with acute cognitive impairment (Nys et al., 2007; N'Gbo N'Gbo Ikazabo et al., 2022). However, research has shown that the extent of impairments depends on the amount of alcohol consumed. Notably, habitual mild-to-moderate alcohol consumption has been linked to reduce the risk, while higher levels of alcohol consumption increase the stroke risk (O'Keefe et al., 2014).

#### 4.2.3 Common neuropsychological assessment scales

Upon keyword analysis, we found that the common neuropsychological assessments such as the mini-mental state examination (MMSE), MoCA, Clinical Dementia Rating Scale Sum of Boxes (CDR-SOB), the Alzheimer’s Disease Assessment Scale–Cognitive Subscale (ADAS-cog), Vascular Dementia Assessment Scale–Cognitive subscale (VDAS-cog), the Clock Drawing Test, the Symbol Digit Modalities Test, the Boston Naming Test, the Neuropsychiatric Inventory Questionnaire, the Loewenstein Occupational Therapy Cognition Assessment, and the Oxford Cognitive Screen are commonly utilized for assessing the baseline and detecting the effectiveness of the treatments for PSCI. Among these assessments, the MMSE and MoCA are currently the most commonly used clinical cognitive screening scales. They are both simple, easy to perform, and appropriate for screening cognitive impairment. However, the MMSE has less sensitivity in identifying mild cognitive impairment (Mitchell, 2009), and it tends to overlook patients with impairments in the language and audiovisual domains and those with concomitant psychiatric symptoms. Meanwhile, the accuracy of the MoCA is affected by the language and motor function, and educational level (Liu et al., 2022). The CDR-SOB, on the other hand, is commonly used to assess the functional impact of cognitive impairment, using semi-structured interviews to evaluate the performance in three cognitive areas (memory, oriented judgment, and problem solving) and three functional areas (community affairs, family and hobbies, and personal care). However, since the development of CDR-SOB mainly focused on AD, it may not be a suitable assessment tool for PSCI. The ADAS-Cog, a global cognitive assessment scale, is considered the gold standard for longitudinal follow-up and clinical drug trials of AD and VCI. It has been widely used as a comprehensive assessment tool for the early diagnosis and evaluation of disease progression and occasionally used for PSCI (Wang and Wang, 2007). However, ADAS-Cog is limited by the subjective nature of some of the assessments, its inability to assess several core deficits (including attention, information processing, and retrieval speed of the stored information), and the specialized training required for administrators (Wesnes, 2008). Impairments in different cognitive domains may have predictive values for changes in an individual’s overall cognitive profile (Stephens et al., 2004). When the overall cognitive assessment scales do not suggest cognitive impairment, multidimensional cognitive assessment scales for each core cognitive domain, such as the Symbol Digit Modalities Test, Boston Naming Test, and Neuropsychiatric Inventory Questionnaire, need to be refined (Wang et al., 2021). The Clock Drawing Test is considered a reliable measure for evaluating the visuospatial and executive function (Palsetia et al., 2018). The Loewenstein Occupational Therapy Cognition Assessment can be used for patients with aphasia. The Oxford Cognitive Screen is focused on assessing five cognitive domains and may be appropriate for patients with aphasia and neglect (Mancuso et al., 2018). Evaluation using multiple scales can improve the validity of treatment evaluation for patients with PSCI.

#### 4.2.4 Animal model

The animal models of PSCI mainly refer to the animal models of VCI or stroke, including the transient global cerebral ischemia model, chronic global hypoperfusion model, and focal cerebral ischemia model, such as the bilateral carotid artery occlusion model, 4-vessel occlusion model, bilateral carotid artery stenosis model, transient or permanent middle cerebral artery occlusion (MCAO), and the microsphere-induced stroke model (Tuo et al., 2021). In addition, focal cerebral ischemia can be induced by the stereotactic injection of endothelin-1 into the bilateral prefrontal cortex of the rat brain (Livingston-Thomas et al., 2015). Among these, the MCAO and microsphere-induced stroke models are most commonly used in PSCI animal studies. The MCAO model causes damage to a small surface area in a fixed infarct location with large stroke volumes and good repeatability. The duration of reperfusion and ischemia is precise and controllable, making it the closest model to simulating human ischemic stroke (Fluri et al., 2015). The model can induce infarct-related cognitive impairment in key brain regions, including the anterior, middle, and posterior arterial perforating lesions, which can affect the thalamus and basal nucleus and cause cognitive impairment. On the other hand, the microsphere-induced stroke model results in multifocal and heterogeneous infarcts, which are manifested by the presence of several areas of damage in the brain and can simulate recurrent stroke (Gerriets et al., 2003). During the last 5 years, the keywords “neurotrophic factor” and “synaptic plasticity” have been the strongest citation burst keywords in PSCI and are the current research trends. Given the role of changes in neurotrophic factors and synaptic plasticity in the pathogenesis and treatment of PSCI, further investigation is needed in animal studies.

### 4.3 Limitations and advantages

Bibliometric analyses utilizing CiteSpace and VOSviewer have the ability to encompass all pertinent literature reports together for analysis. However, they cannot replace systematic reviews due to their methodological limitations. Therefore, this review had some limitations. First, while 355 journals and 1,024 publications were included in our analysis, we only retrieved publications from the SCI-expanded Web of Science Core Collection database, which may have overlooked the contribution of other forms of publications. Second, the search strategy was developed with an effort to include all representations of PSCI, and we broadened the scope of the search to ensure that all literature studies that fit the strategy were retrieved. Subsequently, two researchers specializing in the direction of encephalopathy independently screened the literature studies by reading titles and abstracts based on the inclusion and exclusion criteria and confirmed the results. In case of any discrepancies, a third researcher resolved the disagreements. However, it must be acknowledged that the inclusion and exclusion process still bear the possibility of bias. Third, bibliometric analyses cannot determine the quality of the literature. Hence, low-quality articles may impact the quality of the analysis. Lastly, the results demonstrated that the hotspot analysis focused more on related diseases, risk factors, neuropsychological assessment scales, and animal models. This implies that the mechanisms and treatments of PSCI are not currently the main focus of research and are yet to reach a consensus. Therefore, further research is needed.

Recently, the review published in *NeuroRehabilitation* focused on the bibliometric analysis of PSCI (Ou et al., 2023). However, there were several deficiencies in the overall quality of the article, particularly in their search strategy and literature selection. First, their search strategy was limited to the following keywords (“stroke” OR “apoplexy” OR “Cerebrovascular accident” OR “Cerebrovascular apoplexy”) AND (“cognitive impairment” OR “cognitive dysfunction” OR “cognitive decline”). In SCI-expanded Web of Science Core Collection, because the retrieval of WOS is based on whether the strategy term appears in the title and abstract, a lot of totally irrelevant literature reports having these words (“stroke” OR “apoplexy” OR “Cerebrovascular accident” OR “Cerebrovascular apoplexy” AND “cognitive impairment” OR “cognitive dysfunction” OR “cognitive decline”) mentioned only once in the abstract, and literature reports which focus on PSCI relevant disease so the strategy words are mentioned once as the accompanying symptoms or disease need to be identified, but research contents only focusing on other diseases but not PSCI were included. It may lead to biased results. Second, the inclusion of the term “cerebrovascular accident " without specific inclusion and exclusion criteria in that review resulted in the inclusion of more studies on VCI, which may have further biased the results. Third, it is important to note that the search strategy utilized in that review suggests that the authors believed that the mere co-occurrence of stroke symptoms and cognitive impairment is sufficient for the diagnosis of PSCI. However, this is not accurate as PSCI specifically refers to the causal relationship between stroke events and cognitive impairment. As a result, the search strategy employed in the previous review may have yielded an excessive broad range of articles. Furthermore, the nomenclature of PSCI has only been recently standardized and there are numerous alternative descriptions of PSCI. Therefore, search strategies should take into account all possible alternative descriptions of PSCI. We expanded the scope of the search to ensure that all appropriate literature reports were included based on the inclusion criteria and exclusion criteria. Finally, the previous literature (PMID: 36565073) only covered a period of 10 years, whereas our review covered a span of 20 years, allowing for a more comprehensive analysis of the development of this field. In particular, in this review, based on the analysis of the number of publications, countries, institutions, key words, co-cited authors, and co-cited references, we also added the analysis of hotspots and major findings based on our bibliometric results, including the analysis of PSCI and related diseases, risk factors, common neuropsychological assessment scales, and animal models, in order to better summarize the developments, recent research directions, and hot directions in PSCI research to provide reference for scholars.

## 5 Conclusion

In this review, we have broadened the scope of our literature search to ensure retrieval of publications that may use different terminologies to describe PSCI. We, then, screened the studies by reading titles and abstracts based on the inclusion criteria and exclusion criteria to include all eligible literature studies. Based on the analysis of the number of publications, countries, institutions, co-cited references, and keywords, we clarify the basic information and trends of PSCI research, and highlight the hotspots and major findings in this field. Notably, the number of publications has increased rapidly in the recent 3 years. China, the U.S., and the U.K. dominated in terms of the number of PSCI publications, and the U.S. has a greater impact in the field. Although Chinese universities have published the highest number of articles on PSCI, their international influence still requires further development, with greater emphasis on interinstitutional collaborations to promote further advancements in the field. *Stroke* is the most frequently published and cited journal. Publications that focus on the prevalence, incidence, neuropsychological assessment scales, criteria, and guidelines of PSCI are the most cited references. Presently, “neurotrophic factors” and “synaptic plasticity” have become the research hotspots. Due to the overlap in the neuropathological mechanisms between PSCI, VCI, and AD, these three diseases are often discussed together, and the diagnosis, treatment, and animal models of PSCI are often referred to the experience of AD and VCI. In the future, further studies are necessary to explore the specialized and more applicable medications, scales, and animal models for PSCI.

In 2022, a bibliometric analysis review was focused on VCI. Comparing the results of this review with the bibliometric analysis review of VCI, we can observe some differences. Although the keyword cluster analysis result of VCI and PSCI largely focuses on diagnostic methods and potential risk factors, VCI tends to emphasize blood pressure, oxidative stress, MRI, double blindness, and homocysteine. In contrast, PSCI puts greater emphasis on diffusion kurtosis imaging, MoCA, clinical determinants, activities of daily living, risk factors, and battery. The indicators of researcher attention and emerging research hotspots show that “cerebral small vessel disease,” “beta amyloid protein,” “meta-analysis,” and “mouse model” are the strongest citation burst keywords of VCI, while “neurotrophic factor” and “synaptic plasticity” are the strongest citation burst keywords of PSCI, indicating differences in the recent research study. Additionally, there are differences in the number of publications, countries, institutions, and co-cited references. Therefore, it is still highly meaningful to conduct bibliometric analysis for PSCI, building on the basis of the existing bibliometric analysis of VCI.

In the conclusion of that VCI review by Han et al., the prospect of utilizing bibliometric analysis to explore VCI subtypes was suggested (Han et al., 2022). However, as VCI is a general term that lacks specificity in its mechanism, prognosis, and treatment (van der Flier et al., 2018; Biesbroek and Biessels, 2023), it is essential to focus on specific subtypes, such as PSCI, to derive meaningful results. PSCI, as a subtype of VCI, has a distinct characteristic of relatively fixed brain imaging results and a clear causal relationship between stroke and cognitive function. Therefore, by narrowing the scope of research and exploring the trends and hotspots of PSCI, the results can be made very valuable and not be interfered by other subtypes. At the same time, the timely diagnosis, early intervention, and treatment of PSCI can bring more benefits and more value to the society. Therefore, more research on PSCI is necessary and valuable.

This review helps summarize the literature of PSCI, identify the authoritative and frequently cited literature reports and journals, clarify the current status of PSCI research, and highlight the research hotspots. However, the current research on the mechanisms and treatment of PSCI remains limited. Therefore, we hope that this review has effectively highlighted the research trajectory of PSCI and will lay the foundation for more innovative research in the future.

## Data Availability

The original contributions presented in the study are included in the article/Supplementary Material. Further inquiries can be directed to the corresponding authors.
