# Supplementary material for: Research trends and hotspots of post-stroke cognitive impairment: a bibliometric analysis
Source: Front Pharmacol. 2023 May 30;14:1184830. doi: 10.3389/fphar.2023.1184830 (PMC10267734; doi:10.3389/fphar.2023.1184830)
Supplement: Supplementary file 1 [file DataSheet2.pdf]

Supplementary Material 2

Research trends and hotspots of post-stroke cognitive impairment: A bibliometric analysis

Xiansu Chi[1], Xueming Fan[1], Guojing Fu[1], Yue Liu[1], Yunling Zhang[1*], Wei Shen[1*]

[1] Xiyuan Hospital, China Academy of Chinese Medical Sciences, Beijing, China
*** Correspondence:**
Yunling Zhang
yunlingzhang2004@126.com
Wei Shen
676665709@qq.com

Explanation of the exclusion criteria: The excluded articles consisted of two parts, one part was the literature that was completely unrelated to PSCI through reading the title and abstract. The other part is that in some literatues, research topic has some relationship with PSCI but PSCI is as the concomitant symptom or diseases that need to be identified. In short, the research content only centre on the epidemiology (such as demographic factors, risk factor), mechanisms, diagnosis, pathophysiology, screening , prevention, management or treatment of other PSCI relevant disease but not PSCI. it include literatures: Stroke (publication number=1357), post-stroke delirium (publication number=26), post-stroke aphasia (publication number=34), post-stroke apathy (publication number=12), post-stroke anxiety (publication number=5), post-stroke depression (publication number=103), vascular cognitive impairment (publication number=608), alzheimer's disease (publication number=342) and cognitive impairment due to other causes but not directly due to stroke(n=1205).

Post-stroke cognitive impairment is one of the major complications after stroke,occurs in the 3 to 6 months after stroke onset .Therefore, many articles about stroke will mention the accompanying symptom of PSCI, but the content of the articles is still to discuss the epidemiology, mechanism, imaging, treatment and so on of stroke, and they do not cover PSCI, so this part of literature is excluded. Post-stroke delirium, post-stroke aphasia , post-stroke apathy, post-stroke anxiety and post-stroke depression are mental and psychological disorders that occur after stroke. Although PSCI is mentioned in the abstract, PSCI-related content is not reflected in these articles,so this part of literature is excluded. Vascular cognitive impairment and Alzheimer's disease are the related disease of PSCI, Their similarities and differences have been elaborated in part 4.2.1. Although these literature mentioned PSCI in abstract,they did not focus on the epidemiology (such as demographic factors, risk factor), mechanisms, diagnosis, pathophysiology, screening , prevention, management or treatment of PSCI, so these articles also need to be excluded. At the same time, because PSCI emphasizes the causal relationship between stroke and cognitive impairment, these articles of which cognitive impairment due to other causes but not directly due to stroke also need to be excluded.What need to point out is that if a publication describes the epidemiology(demographic factors, risk factor), mechanisms, pathophysiology, diagnosis, screening, prevention, management or treatment of both PSCI and VCI, it is still included in our 1024 publitions for analysis.
