# Supplementary material for: Research trends and hotspots of post-stroke cognitive impairment: a bibliometric analysis
Source: Front Pharmacol. 2023 May 30;14:1184830. doi: 10.3389/fphar.2023.1184830 (PMC10267734; doi:10.3389/fphar.2023.1184830)
Supplement: Supplementary file 2 [file DataSheet1.pdf]

Supplementary Material 1

Research trends and hotspots of post-stroke cognitive impairment: A bibliometric analysis

Xiansu Chi[1], Xueming Fan[1], Guojing Fu[1], Yue Liu[1], Yunling Zhang[1*], Wei Shen[1*]

[1] Xiyuan Hospital, China Academy of Chinese Medical Sciences, Beijing, China
*** Correspondence:**
Yunling Zhang
yunlingzhang2004@126.com
Wei Shen
676665709@qq.com

1 Supplementary Tables

Table S1 The search strategy

The search strategy for Web of Science core collection	
**Number**	**Search terms**
#1	TS=(("post stroke cognitive impairment") OR ("cognitive impairment after stroke") OR ("post stroke cognitive impairment no dementia") OR ("post stroke dementia"))
	Stroke[MeSH Terms] were searched in Pubmed
	Cerebral HemORrhage[MeSH Terms] were searched in Pubmed
#2	TS=(("stroke") OR ("Cerebrovascular Accident*") OR ("CVA*") OR ("Apoplexy, Cerebrovascular") OR ("Vascular Accident*, Brain") OR ("Cerebrovascular Stroke*") OR ("Stroke*, Cerebrovascular") OR ("Apoplexy") OR ("Cerebral Stroke*") OR ("Stroke*, Cerebral") OR ("Stroke*, Acute") OR ("Acute Stroke*") OR ("Cerebrovascular Accident*, Acute") OR ("Acute Cerebrovascular Accident*") OR ("Cerebral HemORrhage") OR ("HemORrhage*, Cerebrum") OR ("Cerebrum HemORrhage*") OR ("Parenchymal HemORrhage*, Cerebral") OR ("Intracerebral HemORrhage*") OR ("HemORrhage*, Intracerebral")OR ("HemORrhage*, Cerebral") OR ("Cerebral HemORrhage*") OR ("Brain HemORrhage*, Cerebral"))
	Cognitive Dysfunction[MeSH Terms] were searched in Pubmed
	Dementia, Vascular[MeSH Terms] were searched in Pubmed
	Dementia, Multi-Infarct[MeSH Terms] were searched in Pubmed
#3	TS=(("Cognitive Dysfunction") OR ("Cognitive Dysfunctions") OR ("Dysfunction, Cognitive") OR ("Dysfunctions, Cognitive") OR ("Cognitive Impairments") OR ("Cognitive Impairment") OR ("Impairment, Cognitive") OR ("Impairments, Cognitive") OR ("Mild Cognitive Impairment") OR ("Cognitive Impairment, Mild") OR (" Cognitive Impairments, Mild") OR ("Impairment, Mild Cognitive") OR ("Impairments, Mild Cognitive") OR ("Mild Cognitive Impairments") OR ("Mild Neurocognitive DisORder") OR ("DisORder, Mild Neurocognitive") OR ("DisORders, Mild Neurocognitive") OR ("Mild Neurocognitive DisORders") OR ("Neurocognitive DisORder, Mild") OR ("Neurocognitive DisORders") OR ("Mild Cognitive Decline") OR ("Cognitive

	Declines") OR ("Decline, Cognitive") OR ("Declines, Cognitive") OR ("Mental DeteriORation") OR ("DeteriORation, Mental") OR ("DeteriORations, Mental") OR ("Mental DeteriORations") OR ("Dementia, Vascular") OR ("Dementias, Vascular") OR ("Vascular Dementias") OR ("Vascular Dementia") OR ("Vascular Dementia, Acute Onset") OR ("Acute Onset Vascular Dementia") OR ("SubcORtical Vascular Dementia") OR ("Dementia, SubcORtical Vascular") OR ("Dementias, SubcORtical Vascular") OR ("SubcORtical Vascular Dementias") OR ("Vascular Dementia, SubcORtical") OR ("Vascular Dementias, SubcORtical") OR ("Arteriosclerotic Dementia") OR ("Arteriosclerotic Dementias") OR ("Dementia, Arteriosclerotic") OR ("Dementias, Arteriosclerotic") OR ("Binswanger Disease") OR ("Disease, Binswanger") OR ("Chronic Progressive SubcORtical Encephalopathy") OR ("Binswanger Encephalopathy") OR ("Leukoencephalopathy, SubcORtical") OR ("Leukoencephalopathies, SubcORtical") OR ("SubcORtical Leukoencephalopathies") OR ("Encephalopathy, SubcORtical Arteriosclerotic") OR ("Binswanger's Disease") OR ("Binswangers Disease") OR ("Disease, Binswanger's") OR ("Encephalopathy, SubcORtical, Chronic Progressive") OR ("SubcORtical Encephalopathy, Chronic Progressive") OR ("SubcORtical Leukoencephalopathy") OR ("SubcORtical Arteriosclerotic Encephalopathy") OR ("Arteriosclerotic Encephalopathy, SubcORtical") OR ("Arteriosclerotic Encephalopathies, SubcORtical") OR ("Encephalopathies, SubcORtical Arteriosclerotic") OR ("SubcORtical Arteriosclerotic Encephalopathies") OR ("Encephalopathy, Binswanger's") OR ("Binswanger's Encephalopathy") OR ("Encephalopathy, Binswangers") OR ("Encephalopathy, Binswanger") OR ("Encephalopathy, Chronic Progressive SubcORtical") OR ("Dementia, Multi-Infarct") OR ("Dementias, Multi-Infarct") OR ("Multi-Infarct Dementias") OR ("Dementia Multi-Infarct[Title/Abstract]") OR ("Dementia Multi Infarct") OR ("Dementia Multi-Infarcts") OR ("Multi-Infarct, Dementia") OR ("Multi-Infarcts, Dementia") OR ("Dementia, Multiinfarct") OR ("Dementias, Multiinfarct") OR ("Multiinfarct Dementia") OR ("Multiinfarct Dementias") OR ("Multi-Infarct Dementia") OR ("Multi Infarct Dementia") OR ("Lacunar Dementia") OR ("Dementia, Lacunar") OR ("Dementias, Lacunar") OR ("Lacunar Dementias"))
#4	4# 2 AND 3
#5	5# 1 OR 4
